# Trends in the distribution of breast cancer over time in the southeast of Scotland and review of the literature

**DOI:** 10.3332/ecancer.2014.427

**Published:** 2014-05-06

**Authors:** A Aljarrah, WR Miller

**Affiliations:** 1 Breast Unit, Department of Surgery, Sultan Qaboos University Hospital, Al khoud, PO Box 912 PC 111, Muscat, Oman; 2 Edinburgh Breast Unit, Western General Hospital, Edinburgh EH4 2XU, UK

**Keywords:** breast carcinoma, cohorts, quadrants

## Abstract

**Introduction:**

Breast cancer is the most common form of malignancy in Scottish women, and its incidence appears to be increasing with time. It is therefore important to identify factors associated with risk and outcome. Whilst breast cancer occurs equally in the right and left breasts, tumours most commonly affect the upper outer quadrant (UOQ) of the breast. However, there is only limited information as to whether the incidence has changed over time.

**Materials and patients:**

We investigated two cohorts of women diagnosed with breast cancer in the south-east of Scotland between either 1957–1959 or 1997–1999 (i.e., 40 years apart). The earlier cohorts represent 1158 of 1207 women referred to radiation oncologists in the region and the latter group comprised 1477 of about 1600 women referred to the Edinburgh Breast Unit.

**Results:**

Whilst the mean age, menopausal status, and laterality of the patients were similar in both groups, the tumour size and tumour location within the breast were significantly different in the two groups. Thus, there was significant reduction in T stage with year of diagnosis (*p *< 0.0001), the incidence of T1, T2, and T3/4 being 15.6%, 51.9%, and 25.6% in the earlier cohort compared with 49.3%, 36.8%, and 13.7% in the later cohort. The overall distribution within the breast was significantly different by chi-squared analysis ( *p *< 0.0001). In terms of individual quadrants 469 of 1158 (40.5%) tumours were located in the UOQ, whereas in the more recent cohort it was 788 of 1477 (53.4%), this increase in proportion being statistically significant ( *p *< 0.0001). Occurrence in the lower outer quadrant also significantly increased (*p* < 0.028) but was significantly reduced in the upper inner quadrant and centrally (both *p* < 0.0001).

**Conclusion:**

Analysing data on location for each T stage separately showed that the increased incidence in the UOQ with time was apparent for each subgroup. The increased incidence in UOQ tumours over time is therefore not a simple reflection of decreased size between the two time groups. The underlying reason(s) for this change in distribution with time requires further study.

## Introduction

Numerous clinical studies, dating back decades, have shown that the upper outer quadrant (UOQ) of the breast is the most frequent site of carcinoma, but an adequate explanation for this asymmetric occurrence of breast cancer within the breast has never been established. This basic observation has become textbook fact [1] and remains true for countries as different as India [2], the West Indies [3], and Italy [4] and irrespective of race within any one country [5].

Furthermore, the UOQ is not only the most common site for cancer but also, in many benign breast conditions, fibroadenomas, breast cysts [6], and phyllodes tumours [7]. The UOQ is also the most frequent site of male breast cancer [8, 9]. However, it is interesting to note that the reported incidence of breast cancer in the UOQ of the breast appears to rise disproportionately with year of publication. In 1926, 30.9% of breast cancer was reported to be in the UOQ [10] but reports between the years 1947–1967 suggested that the proportion of breast cancer in the UOQ was 43–48% [11–14]. A study in 1994 reported 60.7% of breast cancers in the UOQ [4]. Most of these studies are old. In a recent study in the UK, the distribution was as following, 52.5% of the cases were in the UOQ of the breast [15].

However, the factors involved remain contentious, but it has been suggested that tissue mass is an important contributor to asymmetry in cancer incidence [16]. This has been attributed to more epithelial cells on the left side of the body due to preferential vascular supply to the left side of the body during intrauterine cardiac development [17, 18].

In a recent study done in Nottingham, the quadrant from which 746 consecutive breast core biopsies were reported as normal, benign, or malignant was recorded. The distribution in the breast of normal, benign, and malignant results were comparable. In particular, the proportion of core biopsies from the UOQ reported as normal 67%, benign 57%, or malignant 62% were similar. This result supports the hypothesis that the high proportion of UOQ carcinomas of the breasts is a reflection of the greater amount of breast tissue in this quadrant [19]. An alternative explanation of this could simply be that the UOQ is the local area in vicinity to the axilla where deodorants and antiperspirants are applied. Since they are applied in large amounts, they may simply penetrate through the skin of the local area and mimic the effect of oestrogen in the breast tissue [15].

The importance of tumour location has an important role in the prognosis of breast cancer as it has been proved that early breast cancers situated in central/internal quadrants have a worse prognosis compared with those in lateral quadrants, in terms of distant metastases and survival [20]. In another study [21, 22], medial location was associated with a 50% excess risk of systemic relapse and breast cancer death compared with lateral location. Another study from Italy [23] showed statistically significant differences for patients with medial tumours versus those with non-medial tumours in disease-free survival. However, limited data are available as to whether there has been a change in the distribution of cancer within the breast over time in relation to tumour size, menopausal status, and age. The present paper was designed to address this issue by studying two series of patients diagnosed in the Edinburgh Breast Unit over four decades apart, to determine the site of cancer within the breast of two separate cohorts of women referred within the same geographical area, and whether site was related to other clinico-pathological features of the disease.

## Materials and methods

### Patients

Two cohorts of women presenting with breast cancer in the southeast of Scotland were studied: (i) 1158 out of almost 1400 patients diagnosed with breast cancer between 1957 and 1959, these are all patients who were referred to an oncologist for further treatment, the site of the tumour's location was documented on the clinical notes in the patient's file and (ii) 1477 out of almost 1600 patients diagnosed with breast cancer between 1997 and 1999 and referred to the Edinburgh Breast Unit. The cases were selected on the basis of being able to retrieve the following details from files: confirmation of histological breast cancer, patient age and menopausal status, and tumour site, size, and laterality (patients with bilateral tumours and males were excluded from this paper). These data were recorded prospectively on a proforma which was subsequently computerised.

### Tumour location

Clinically, the breast can be divided into five quadrants (upper outer, lower outer, upper inner, lower inner, and central). This was utilised in this study to categorise tumour location, corresponding to UOQ (Q1), lower outer quadrant (LOQ, Q2), upper inner quadrant (UIQ, Q3), lower inner quadrant (LIQ, Q4), and central (Q5). Big tumours which occupied more than one quadrant or small tumours located on the interface between two quadrants, e.g., at 12, 3, 6, and 9 o'clock, were classified in a single category (Q6).

### Definition of menopausal status

Patients who were menstruating or were within three years of their last menstrual period were classified as premenopausal; those beyond three years of their last menopausal period were regarded as being postmenopausal [24]. In the cohort 1957–1959, out of 1158 patient 54 did not have their menopausal status recorded, this leaving 1104 patients. In the cohort 1997–19999, all 1477 patients had their menopausal status recorded.

### T stage

T stage of the cohort 1957–1959 was available only for tumour pathological size; there were no records on the lymph nodes status and metastasis. As a result of this, only pathological tumour size was available for comparison between the two cohorts. Pathological tumour size in the cohort 1957–1959 was recorded for 1093 out of 1158 (65 patients did not have their pathological size recorded). In the cohort 1997–1999 all 1447 patients had their pathological sizes recorded. Due to small numbers of cases in T3 and T4 they were put together as T3/4.

## Results

Both groups of patients were selected on the basis of being female, having a diagnosis of breast carcinoma, unilaterality of disease and clear documentation of site of tumour within the breast. This provided 1158 patients in 1957–1959 (out of a total of 1208 referred to the radiation oncology in Edinburgh) and 1477 in 1997–1999 (out of almost 1600 patients referred to the Edinburgh Breast Unit). The demographic characteristics of patients and their tumours are shown in Table 1. The mean age at diagnosis for the 1957–1959 cohorts (57.7 years) was not significantly different from that of the 1997–99 cohorts (61.6 years). The menopausal status was known for all patients in the 1997–1999 cohort, but was missing for 54 cases in the earlier group. At diagnosis, 27.1% of women in the first cohort were premenopausal compared with 28.8% for the 1997–1999 cohorts. This change in the proportion of premenopausal women was not statistically significant by the Chi-square test ( *χ *^2^ = 0.88, *p* = 0.35).

### Tumour location in the breast

The sites of the tumours within the breast are summarized in Table 2 for both groups of women. The distribution in the breast was significantly different between 1957–1959 and 1997–1999 ( *χ*^2^ = 100.288, *p* = 0.0001).

The highest incidence of tumour was in the UOQ of the breast. Almost half of the cases were found in this location irrespective of the time of diagnosis (Table 2). This increase in proportion was statistically significant (*χ*^2^ = 42.45, *p *< 0.0001). The incidence of tumours in the LOQ has increased too, statistically significant (*χ*^2^ = 4.83, *p *= 0.028). In contrast to the UOQ and LOQ, the UIQ decreased in incidence and was statistically significant ( *χ*^2^ = 26.05, *p* < 0.0001).

The incidence of tumours in LIQ was similar in both groups, was not statistically significant ( *χ*^2^ = 0.632, *p *= 0.43), the central location decreased in the incidence this difference was statistically significant (*χ*^2^ = 40.88, *p *< 0.0001). Q6 category showed a reduction in incidence, was statistically significant (*χ*^2^ = 23.38, *p* < 0.0001).

### Location of tumour in the breast according to T stage

Because there were statistically significant differences in the patient cohorts with regard to T stage, it was of interest to determine whether this factor influenced changes in tumour location with time. These data are shown in Table 3.

The difference in tumour location between 1957–1959 and 1997–1999 was apparent in all T stages, the difference being *p *< 0.0001 in all cases. In terms of individual quadrants, consistent statistical significances were seen for each T stage for UOQ. The increased incidence in UOQ tumours over time is therefore unlikely to be a reflection of decreased size. Where statistically significant differences were apparent between time groups for other zones, similar trends were also observed after subdivision into T stage; some did not reach statistical significance, probably on account of the small number of cases. The Q6 zone was unusual in that there was an increasing incidence in 1997–1999 in T1 and T2 and a statistically significant decrease with T3/4.

## Discussion

The present paper has shown that over the last four decades there has not been an apparent change in menopausal status or in the laterality of patients with breast cancer, which is conflicting with other results, but there were significant changes for the size and distribution of breast cancer. It is important to consider the possible reason for these changes.

Thus, even if the observations are specific to Edinburgh, the characteristics of patients and tumours in this location have changed. It is possible that the difference results from the selection process used to identify patients during the second time period, but the distribution of cancers right versus left and menopausal status did not change and there is no reason why patients with breast cancer or those with UOQ lesion should have had changed incidence. This result is different compared with other published studies mentioned in the introduction.

Whilst, the initiation of the screening programme in Edinburgh in 1991 will have had an impact on the population of women presenting with breast cancer, it is more likely to have reduced the proportion of premenopausal women, screening being offered to mainly postmenopausal women. The most significant difference in the cancers between the cohorts was the increase in tumour size. Similar changes over time have been previously reported. This is likely to be in large part because of better education with increasing breast awareness and the screening programme. The present paper has confirmed previously reported findings that the distribution of breast cancer is not even throughout the breast but is higher in the UOQ. The paper has also demonstrated that the preponderance of cancer in the UOQ is increasing with time. In the most recent time period almost half of cases were found in the UOQ irrespective of the laterality of the disease. The increases trend was significant (*p *< 0.0001). There was an increase in the likelihood of finding a cancer in the LOQ. The increased incidence in these quadrants was particularly seen in premenopausal women but was apparent in each T stage, unlike another study where there was increment in all quadrants. The incidence of UOQ tumours was higher in premenopausal women in both cohorts (*p *< 0.0001). The reason for the higher incidence of breast cancer at this site is ascribed to a greater proportion of breast tissue in the UOQ [1] and some changes in lifestyle [15].

In this paper, the change in the quadrant was correlated to the size of the tumour and the menopausal status, this will give a strong result with regards to bias with the site of the tumour in the quadrant.

The ability to reduce breast tumour growth through manipulation of oestrogen action has played a central role in the endocrine therapy of breast cancer [25], but little consideration has been given to the potential interaction of the presence of oestrogenic chemicals in the human breast on the effectiveness of this therapy in individual patients. Interestingly, the UOQ is not only the most common site of the tumour in cancer but also of the abnormalities in benign breast conditions, including fibroadenoma, breast cysts [6], and phyllodes tumour [1, 30].

Explanations for an increase of tumours in the UOQ include the possibility that agents administered topically to the axilla might gain access to the breast and be responsible for the initiation/promotion of tumours at that site. Interestingly compounds in deodorants, such as parabens, have been reported to have the ability to penetrate the skin and have oestrogenic activity [26]. Whether this might promote tumour growth remains a matter of conjecture.

Since antiperspirants act by blocking sweat ducts [27], and breast cysts result from blocked breast ducts [2], it is possible that breast cysts could also arise from repetitive trauma to the ducts in this area. Studies of the relation between cysts and breast cancer have conflicting results. Some studies showed women with breast cysts are at an increased risk of breast cancer, especially at younger ages [28, 29]. Phytoestrogens are used increasingly in cosmetics designed for application around the human breast. There is an increasing trend towards addition of Aloe Vera into personal care products, and the constituent anthraquinones are known to possessoestrogenic properties [30]. Cosmetic chemicals, such as aluminium salts [31], cyclosiloxanes, and triclosan are already known to have DNA damaging properties as well as oestrogenic activity [32, 33]. The alkyl esters of p-hydroxybenzoic acid (parabens) are added in concentrations of up to 0.8% as preservatives to thousands of cosmetic products, which mimic oestrogen in breast tissue [8, 15] and have been detected in human breast tumour tissue at an average concentration of 20 ng/g tissue [34]. The constant use of bras (particularly of under-wired which constricts breast tissue and lymphatics mostly in the outer quadrants by the very nature of its design) for long periods might influence lymphatic flow from the breast, this might be a cofactor with other factors in traumatizing tissues in the UOQ of the breast where the wire has the most pressure point.

Axillary hair is now frequently removed by different means and is currently performed more frequently than was done four decades ago. This potentially causes repetitive trauma to the axilla and neighbouring outer quadrants. The accepted explanation for the disproportionate incidence of breast cancer in the UOQ of the breast is that this region of the breast contains a greater proportion of the epithelial tissue [19], which is the target site for breast cancer. However, evidence for this explanation is lacking and seems to be anecdotal in origin. If this trend to increasing incidence of breast cancer in the UOQ is a function of time and is not a reflection of different study populations, then this would question the explanation for high incidence of breast cancer in the UOQ as being due solely to the presence of more epithelial tissue in that region. However, further studies need to be done to assess the cause of this disproportion especially for epidemiology prognosis. Other epidemiological factors which may have changed between the two time cohorts and which may influence the risk of breast cancer include the length of pill usage, use of HRT, late first pregnancy, number of offspring/family size and long gap between pregnancies, alcohol, and cigarette smoking. There is no obvious reason why these particular factors should influence tumour location, but it might influence breast cancer frequency and tumour size. Further studies need to be carried out to determine what might be the cause of this change in the distribution of breast cancer. It should also be noted that changes over time in breast cancer do not only affect location. For example, there is some circumstantial evidence that temporal changes may be occurring in laterality. Many studies indicate that the left breast is more prone to development of cancer than the right breast in both female [1, 11, 12, 18, 35] and male [17, 18] breast cancer. However, in a more recent American publication describing patients studied between 1973 and 1998, the numbers of right-sided and left-sided were roughly equal [36]. Interestingly, the most common location for tumours was the UOQ of the breast. The stage and histology of breast cancer has also changed. As was also shown in the present paper, more early breast cancer is being seen now as compared with in the past [37]. Similarly, while ductal carcinoma incidence rates remained essentially constant from 1987 to 1999, lobular carcinoma rates increased steadily [38, 39]. This may be because HRT use has increased steadily from the 1970s to the 1990s [40–44]. Beyond their aetiologic importance these results also have clinical significance because invasive lobular carcinoma (ILC) and invasive ductal carcinoma (IDC) have different clinical features. For example, ILC is more likely to be hormone receptor-positive [45] and to have a better prognosis than IDC [46].

In view of the fact that breast cancer increasing worldwide and the distribution in the UOQ has also increased, this is considered to be new data on cancer distribution in the breast, the cause of which needs further research and may be due to many factors.

## Figures and Tables

**Table 1.** The demographic characteristics of patient in two cohorts.

	1957–59	1997–99	*P*values
**Mean age (years)**	57.7	61.6	
**Menopausal status**			0.35
**Pre**	299 (27.1%)	426 (28.8%)	
**Post**	805 (72.9%)	1051 (71.2%)	
**T stage**			0.0001
**1**	181 (16.6%)	729 (49.4%)	
**2**	625 (57.2%)	545 (36.9%)	
**¾**	287 (26.2%)	203 (13.7%)	
**Laterality**			
**L**	585 (50.5%)	753 (51%)	
**R**	573 (49.5%)	724 (49%)	

T stage was known for all patients in the 1997–99 cohorts, but was missing in 65 cases in the earlier cohort. In terms of T stage, there was a highly significant decrease, ( *χ*^2^ = 24.6, *p* < 0.0001) between 1957–59 and 1997–1999; 181 (16.5%) were T1, 625 (57.2%) T2, and 287 (26.2%) T3/4 patients in cohort 1957–1959, as compared with 729 (49.3%) T1, 545 (36.8%) T2, and 203 (13.7%) T3/4 in cohort 1997–1999.

**Table 2.** The site of breast cancer in different quadrants in two cohorts 1957–59 and 1997–99 (*χ*^2^ = 100.288, *p*= 0.0001).

Site	1957–1959	1997–1999
**UOQ**	469 (40.5%)	788 (53.4%)
**LOQ**	86 (7.4%)	147 (10%)
**UIQ**	221 (19.1%)	175 (11.8%)
**LIQ**	62 (5.4%)	91 (6.2%)
**Central**	145 (12.5%)	69 (4.7%)
**Q6**	175 (15.1%)	207 (14.0%)
**TOTAL**	1158	1477

**Table 3.** The incidence of tumours in the breast zones in each T stage category.

	UOQ	LOQ	UIQ	LIQ	Central	Q6	Total
T1 1957–1959	75 (41.4%)	24 (13.3%)	43 (23.8%)	18 (9.9%)	13 (7.2%)	8 (4.4%)	**181 ** (16.6%)
T1 1997–1999	380 (52.1%)	74 (10.2%)	87 (11.9%)	53 (7.3%)	33 (4.5%)	102 (14.0%)	**729 ** (49.4%)
Stats overall comparison *χ*2 = 33.15, *p* < 0.0001							
T2 1957–1959	289 (46.2%)	43 (6.9%)	134 (21.4%)	36 (5.8%)	70 (11.2%)	53 (8.5%)	**625 ** (57.2%)
T2 1997–1999	290 (53.2%)	50 (9.2%)	70 (12.8%)	29 (5.3%)	17 (3.1%)	89 (16.3%)	**545 ** (36.9%)
Stats overall comparison *χ*2 = 67.47, *p* < 0.0001							
T3/4 1957–1959	81 (28.2%)	14 (4.9%)	32 (11.1%)	6 (2.1%)	46 (16.0%)	108 (37.6%)	**287 ** (26.2%)
T3/4 1997–1999	118 (58.1%)	23 (11.3%)	18 (8.8%)	9 (4.4%)	19 (9.3%)	16 (7.8%)	**203 ** (13.7%)
Stats overall comparison *χ*2 = 81.04, *p* < 0.0001							
