# Erratum: Trends in the distribution of breast cancer over time in the southeast of Scotland and review of the literature

**DOI:** 10.3332/ecancer.2014.439

**Published:** 2014-06-26

**Authors:** A Aljarrah, WR Miller

**Affiliations:** Edinburgh Breast Unit, Western General Hospital, Edinburgh EH4 2XU, UK

## Abstract

*e*cancer **8** 427 (2014)

WR Miller was not involved in and did not approve submission of this article.

